# Women Have Higher Protein Content of β-Oxidation Enzymes in Skeletal Muscle than Men

**DOI:** 10.1371/journal.pone.0012025

**Published:** 2010-08-06

**Authors:** Amy C. Maher, Mahmood Akhtar, Jerry Vockley, Mark A. Tarnopolsky

**Affiliations:** 1 Department of Medical Science, McMaster University, Hamilton, Ontario, Canada; 2 Department of Pediatrics and Medicine, McMaster University, Hamilton, Ontario, Canada; 3 University of Pittsburgh School of Medicine (Pediatrics) and Graduate School of Public Health (Human Genetics), Pittsburgh, Pennsylvania, United States of America; Texas A&M University, United States of America

## Abstract

It is well recognized that compared with men, women have better ultra-endurance capacity, oxidize more fat during endurance exercise, and are more resistant to fat oxidation defects i.e. diet-induced insulin resistance. Several groups have shown that the mRNA and protein transcribed and translated from genes related to transport of fatty acids into the muscle are greater in women than men; however, the mechanism(s) for the observed sex differences in fat oxidation remains to be determined. Muscle biopsies from the *vastus lateralis* were obtained from moderately active men (N = 12) and women (N = 11) at rest to examine mRNA and protein content of genes involved in lipid oxidation. Our results show that women have significantly higher protein content for tri-functional protein alpha (TFPα), very long chain acyl-CoA dehydrogenase (VLCAD), and medium chain acyl-CoA dehydrogenase (MCAD) (P<0.05). There was no significant sex difference in the expression of short-chain hydroxyacyl-CoA dehydrogenase (*SCHAD*), or peroxisome proliferator activated receptor alpha (*PPARα*), or *PPARγ*, genes potentially involved in the transcriptional regulation of lipid metabolism. In conclusion, women have more protein content of the major enzymes involved in long and medium chain fatty acid oxidation which could account for the observed differences in fat oxidation during exercise.

## Introduction

A number of studies have shown that women have a lower respiratory exchange ratio (RER) compared with men, indicating higher lipid oxidation and lower carbohydrate (CHO) oxidation during moderate intensity endurance exercise [1]–[11]. Furthermore, women have higher whole body lipolysis and greater skeletal muscle uptake of plasma free fatty acids (FFA) [12], higher intramyocelluar lipid (IMCL) content [10], [13]–[15] and higher net IMCL utilization [10], [16], as compared to men during endurance exercise. The potential mechanism(s) of such sex differences have only recently been evaluated in human based research, but include sex based differences in gene expression at the RNA and protein level, sex differences in response to an acute bout of endurance exercise, and/or hormonal regulation of pathways involved in metabolism [11], [17]–[20]. Several studies have shown that sex differences alone are greater predictors of substrate selection than are age, menstrual cycle phase, 17β-estradiol supplementation, endurance and strength training effects on mRNA content [20]–[23].

Skeletal muscle from women shows higher mRNA and protein content of fatty acid transporter (FAT/CD36) [18], and hormone sensitive lipase (HSL) [10] than in men. Women also have higher mRNA expression of lipoprotein lipase (LPL) [18], membrane fatty acid transport protein 1 (FATm) [17], plasma membrane fatty acid binding protein (FABPpm) [18], CPT I [24], trifunctional protein-β (TFPβ) [19], peroxisome proliferator activated receptor-α and δ (PPARα, PPARδ), cytosolic fatty acid binding protein (FABPc), sterol regulatory element binding protein –1c (SREBP-1c), and mitochondrial glycerol phosphate acyltransferase (mtGPAT) [23] than men. Taken together, these results suggest that women are transcriptionally programmed for greater fatty acid transport into the skeletal muscle, β-oxidation, and IMCL synthesis than men.

Lipid oxidation occurs in the mitochondria through β-oxidation. Fatty acids (FAs) enter the mitochondria by transporters CPTI, CPTII and FAT/CD36 [25] or diffusion, depending on chain length. Women have significantly higher mRNA for CPTI than men; however, there is no significant sex difference in CPT1 protein or activity [24]. Recent evidence suggests that there are FAT/CD36 transporters on the mitochondrial membrane [25]–[26] and although sex differences in the mitochondria specific population have not been examined, women have significantly greater FAT/CD36 protein in whole muscle homogenate compared with men [18], which may allow for greater mitochondrial uptake of long chain fatty acids (LCFAs) in women.

There are four major enzyme activities in the β-oxidation pathway; acyl-CoA dehydrogenases, enoyl-CoA hydrateses, 3-hydroxyacyl-CoA dehydrogenases, and 3-ketoacyl-CoA thiolases [27]. At least three enzymes with activities toward different substrates perform the first reaction, very long-, medium-, and short chain acyl-CoA dehydrogenases (VLCAD, MCAD, and SCAD, respectively). The second through fourth reactions for long chain substrates are found on the mitochondrial trifunctional protein (TFP), a heteroctamer made of each of α and β subunits. Sex based differences of β-oxidation enzymes have only recently been studied. The mRNA content of β-hydroxyacyl-CoA dehydrogenase [β-HAD, also known as short-chain β-OH acyl-CoA dehydrogenase (SCHAD)] is greater in women compared with men; however, there is no sex difference in β-HAD activity [11]. Similarly, women have higher levels of acyl-CoA acyltransferase 2 (ACAA2) mRNA with no sex specific difference in protein content [19]. Both β-HAD (SCHAD) and ACAA2 are responsible for short chain FA oxidation, but long chain- and medium chain- acyl-CoA oxidation has not been compared between the sexes. Due to the convincing evidence that women have greater whole body FA oxidation and uptake of FAs during exercise than men, we hypothesized that there would be sex differences in the protein capacity for LCFA oxidation in human skeletal muscle.

The purpose of this study was to evaluate the effect of sex on the mRNA expression and protein content of genes involved in β-oxidation (*VLCAD*, *MCAD*, *TFP-α*, *SCHAD*) and potential regulators of these proteins (*PPARα*, *and PPARγ*) in human skeletal muscle. We specifically hypothesized that the mRNA and protein content for the genes involved in lipid metabolism would be higher in women than men.

## Materials and Methods

The study was approved by the McMaster University Hamilton Health Sciences Human Research Ethics Board and conformed to the Declaration of Helsinki guidelines.

### Subjects

Twenty-three, young (22±2 y) healthy, non-smoking, non-obese, recreationally active men (n = 12) and women (n = 11) participated in the current study. The subject characteristics are described in Table 1. Men and women were matched based on peak O_2_ consumption (VO_2peak_) expressed as milliliters per kilogram fat-free mass per minute. Women were in the follicular phase of their menstrual cycle (day 3–13). All subjects gave informed written consent prior to participation.

**Table 1:** Subject characteristics.

	Men (n = 12)	Women (n = 11)
Age (yr)	21±1	20±1
Weight (kg)	83±5	66±1 a
Height (cm)	181±3	167±1 a
Body Fat (%)	17±2	32±1 b
Fat Mass(kg)	14±2	20±6 b
Fat Free Mass (kg)	66±3	42±1 a
VO_2_peak (ml*kg body wt-1*min-1)	44±2	39±2 a
VO_2_peak(ml*kg FFM-1*min-1)	56±2	60±3

Data are means ±SE. a; significantly lower in women P≤0.05, b; significantly higher in women P≤0.05.

### Study design

At least one week prior to testing, subjects completed a whole body DEXA scan and progressive VO_2peak_ test on a stationary electronically braked cycle ergometer and a computerized open-circuit gas collection system (Moxus Modulator VO_2_ system with O_2_ analyzer S-3A/I and CO_2_ analyzer CD-3A, AEI Technologies Inc., Pittsburgh, PA) as previously described [1], [5], [7]–[8]. Subjects completed diet records for three days leading up to testing (no significant difference in percent fat, carbohydrate or protein intake between men and women, data not shown), and did not exercise 48 h before testing. Subjects were asked to refrain from eating after 2100 h the day before testing and were given a defined formula drink (Ensure Plus®, Abbott Laboratory Inc., Saint-Laurent, Quebec, Canada) 2 h before baseline blood and muscle biopsies were acquired. All samples were taken in the morning. Blood was collected and resting glucose and lactate levels were determined using a blood-gas analyzer (Radiometer ABL800 FLEX, Copenhagen, Denmark) (no significant difference between men and women, data not shown). Serum estradiol levels were evaluated using a human estradiol ELISA kit; Fertigenix-E2-EASIA (Biosource Europe S.A, Nivelles, Belgium) (no significant difference between men and women, data not shown). Muscle biopsies (∼150 mg) were taken from the *vastus lateralis* muscle before 90 min of stationary cycling at 65% of their VO_2peak_. The muscle was rapidly placed in an RNase-free polyethylene tube, flash-frozen in liquid nitrogen, and stored at −80°C until being processed for analysis. Respiratory measures (oxygen uptake, expired CO_2_, and RER) were taken using the computerized open-circuit gas collection system, and fat and glucose oxidation rates were determined using the non-protein respiratory quotient [28], and percentage fat and glucose utilized was determined as previously described [2]. Respiratory measures were taken from 10–15 min, 30–40 min and 60–70 min during the ride. The subjects maintained the same pedaling cadence (rpm) throughout the ride. At each time point the wattage was adjusted (if needed) so that the subjects maintained 65% of their V0_2peak_. Data used to express the respiratory measures (oxygen uptake, expired CO_2_, and RER) were taken from the 30 and 60 min time points and averaged together to represent RER over 30 min in the middle of the exercise bout. These data points ensure the subjects were at a steady state.

### Preparation of RNA

Fifty milligrams of human muscle was used to isolate total RNA using mirVana™ RNA isolation kit (Ambion Inc., Austin, TX #AM1561). In brief, muscle tissue was homogenized in lysis/binding buffer in a glass homogenizer. The RNA was extracted organically using acid-phenol∶chloroform and ethanol precipitation. Final isolation was done using provided filter cartridges. RNA was eluted in nuclease-free water and quantity and quality of RNA was assessed using a NanoDrop Spectrophotometer (Thermo Scientific, Wilmington, DE). Measurements were done in duplicate and had an average coefficient of variation (CV) of <10%. The average purity (OD_260_/OD_280_) of the samples was >1.8.

### TaqMan® real-time RT-PCR

First-strand cDNA synthesis from 100 ng of total RNA was performed with random primers using a High Capacity cDNA Reverse Transcription Kit (Applied Biosystems Inc., Foster City, CA Cat#4368814) according to manufacturer's directions. Gene expression was quantified using 7300 Real-time PCR System (Applied Biosystems Inc., Foster City, CA) and SYBR® Green chemistry (PerfeC_T_a SYBR® Green Supermix, ROX, Quanta BioSciences, Gaithersburg, MD) as previously described [29]. Specific primers to each target mRNA (Table 2) were designed based on the cDNA sequence in GenBank with MIT primer 3 designer software. Thermal dynamics was optimized through calculating delta G with Analyzer of Oligo. Primer specificity was checked using Blast and RT-PCR dissociation curves. All samples were run in duplicate on a 96-well plate. Each target gene was run in parallel with human β2- microglobulin (β2-M) as an internal standard with RNA- and RT-negative controls.

**Table 2:** Real-time PCR primer sequences.

Gene	Accession	Forward primer (5′-3′)	Reverse primer (5′-3′)
VLCAD	BC012912	gtggccgctttctgtctaac	ccttcgttcgaaacctagtc
MCAD	AF251043	tgccagagaggaaatcatcc	tctcggacccttgaaccaaa
TFPα	BC009235	gagttgacccgaagaagctg	aaccacacctacatcgcttt
SCHAD	BC000306	cgttgtccacagcacagact	gacctgttcaaacgacgact
β2M	NM_004048	ggctatccagcgtactccaa	gatgaaacccagacacatagca

### Immunoblotting analysis

Thirty mg of tissue were used for protein content analysis. Muscle tissue was homogenized in a phosphate lysis buffer; 50mM K_2_HPO_4_, 1mM EDTA, pH7.4, 0.1mM DDT, PhosSTOP (Roach Diagnostics, Mannheim, Germany), Protease inhibitor cocktail tablets (Roach). Protein concentrations were calculated by Bradford assay (Bio-Rad Laboratories, Hercules, CA) and equal amounts of protein were boiled in Laemmli buffer, resolved by SDS-PAGE, transferred to nitrocellulose paper or PVDF and immunoblotted with desired antibodies. Primary antibodies; VLCAD and MCAD (Dr. J. Vockley), HADHA (TFP-α) (Protein Tech Group, Chicago, IL), SCHAD (Abcam, Cambridge, MA), PPARα (Santa Cruz Biotechnology, Santa Cruz, CA), PPARγ (Cell Signaling Technology, Danvers, MA), actin (Abcam) and citrate synthase (CS) (Dr. B. Robinson, Sick Kids Hospital, Toronto). There were no sex differences in protein content of citrate synthase (mitochondrial content) or actin (total myofibrillar and cytosolic protein loading). Actin and ponceau were used as loading controls. Secondary antibodies conjugated to horseradish peroxidase (Amersham Bioscience, UK) and specific antibody binding was detected using the chemiluminescence detection reagent ECL Plus (Amersham BioScience, UK). Scanned films were analyzed using ImageJ 1.40 software (Wayne Rasband National Institute of Health, USA).

### Statistical Analysis

Statistical analyses of mRNA expression of the genes were performed by subtracting the linear CT (2^−CT^) of the house keeping gene (β2-M) from the linear CT of gene of question [30]. Statistical analysis of gender differences in mRNA and protein content (±SE) were calculated using a Student's t-test. Correlation analysis (Pearson's) was used to determine the relationship between RER and protein content in all subjects. Statistical significance was set at α≤0.05.

## Results

### Subject Characteristics

Women had significantly lower body weight, height, fat free mass (FFM), and VO_2peak_ per kilogram body weight (P≤0.05) compared with men (Table 1). When VO_2peak_ was expressed relative to FFM there was no significant difference between men and women. During exercise women had a significantly lower RER (0.87±0.02) than men (0.91±0.01) (P≤0.05) (Table 3).

**Table 3:** Substrate oxidation in men and women cycling at 65% VO_2peak_.

	Men	Women
RER	0.91±0.01	0.87±0.02 *
Fat oxidation (g/min)	0.36±0.06	0.38±0.05
Glucose oxidation (g/min)	2.36±0.14	1.31±0.12 *
Fat oxidation (%)	29.6±3.5	45.0±4.9 *
Glucose oxidation (%)	70.4±3.5	55.0±4.9 *

Data are means ±SE, RER; respiratory exchange ratio, *P≤0.05.

### Sex differences in key enzymes of the β-oxidation pathway

#### VLCAD

**Figure 1:**
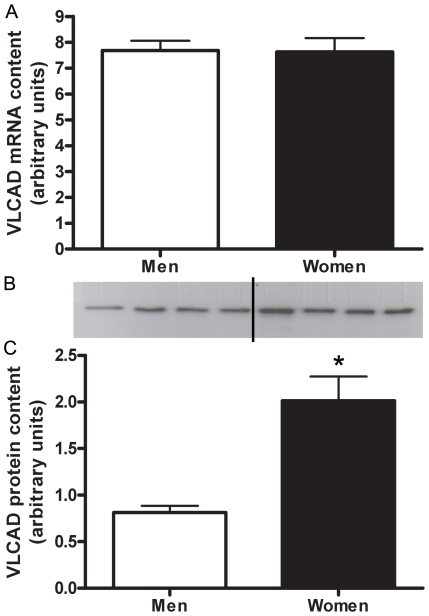
VLCAD protein content is higher in women than men. Differences in VLCAD mRNA content between men and women shown by Real time RT-PCR, adjusted to β2-M mRNA (A). Protein content of VLCAD in skeletal muscle of men and women, adjusted to CS (B,C). Representative western blot (B) Lanes 1–4 are men, lanes 5–8 women. N = 12 men and 11 women. *P≤0.05.

There was no sex difference in the mRNA abundance of VLCAD (Figure 1 A). Women had a 2.5±0.4 fold (P = 0.001) higher protein content of VLCAD compared with men (Figure 1B, C). VLCAD protein content had a significant correlation with RER (r^2^ = 0.35, P = 0.001).

#### MCAD

**Figure 2:**
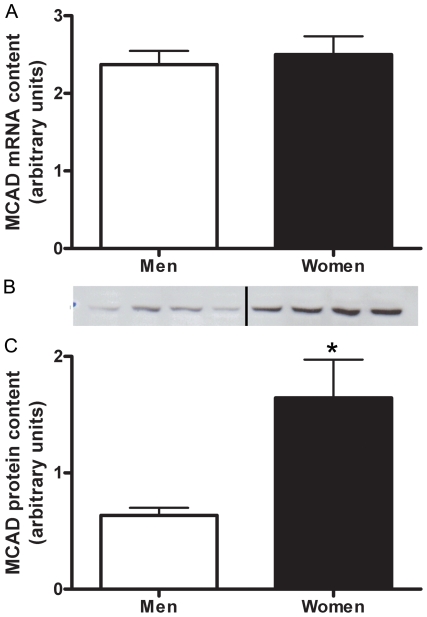
MCAD protein content is higher in women than men. Differences in MCAD mRNA content between men and women shown by Real time RT-PCR, adjusted to β2-M mRNA (A). Protein content of MCAD in skeletal muscle of men and women, adjusted to CS (B,C). Representative western blot (B) Lanes 1–4 are men, lanes 5–8 women. N = 12 men and 11 women. *P≤0.05.

There was no sex difference in the mRNA abundance of MCAD (Figure 2 A). Women had a 2.6±0.6 fold (P = 0.007) higher protein content of MCAD than men (Figure 2 B, C). MCAD protein content had a significant correlation with RER (r^2^ = 0.21, P = 0.01).

#### TFP- α

**Figure 3:**
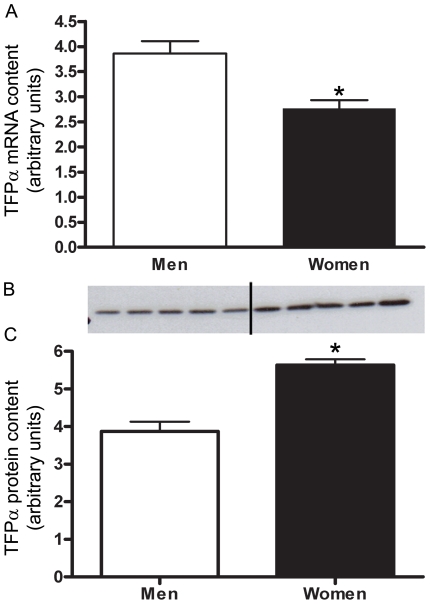
TFPα protein content is higher in women than men. Differences in TFPα mRNA content between men and women shown by Real time RT-PCR, adjusted to β2-M mRNA (A). Protein content of TFPα in skeletal muscle of men and women, adjusted to CS (B,C). Representative western blot (B) Lanes 1–5 are men, lanes 6–10 are women. N = 12 men and 11 women. *P≤0.05.

Women had a significantly lower abundance (−1.4±0.1 fold p = 0.004) of TFP-α mRNA compared with men (Figure 3A). Women had a 1.5±0.1 fold (P<0.001) higher protein content of TFP-α than men (Figure 3B, C). TFP-α protein content had a significant correlation with RER (r^2^ = 0.23, P = 0.02).

#### SCHAD

**Figure 4:**
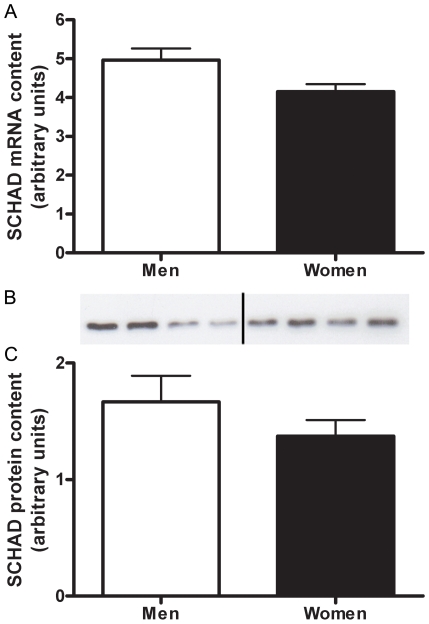
SCHAD protein content is equal in women and men. SCHAD mRNA content between men and women shown by Real time RT-PCR, adjusted to β2-M mRNA (A). Protein content of SCHAD in skeletal muscle of men and women, adjusted to CS (B,C). Representative western blot (B) Lanes 1–4 are men, lanes 5–8 women. N = 12 men and 11 women.

There was no sex difference in the mRNA abundance or protein content of SCHAD (Figure 4). There was no correlation between SCHAD protein content and RER (r^2^ = 0.0004, P = 0.46)

### Regulation of lipid metabolism

**Figure 5:**
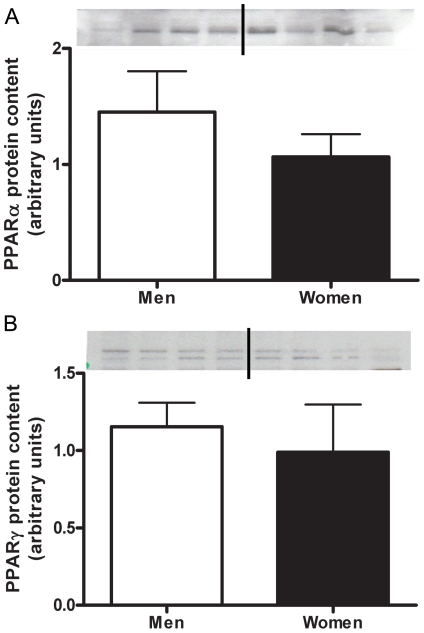
No sex differences in PPARα or PPARγ protein content. Protein content of PPARα (A) and PPARγ (B) in skeletal muscle of men and women, adjusted to actin. Representative western blots; lanes 1–4 are men, lanes 5–8 women. N = 12 men and 11 women.

There was no sex difference in the protein content of PPARα and PPARγ (Figure 5); two transcription factors involved in the regulation of mitochondrial FA oxidation proteins. There was no correlation between either PPARα or PPARγ protein content and RER (r^2^ = 0.06, P = 0.17; r^2^ = 0.004, P = 0.4)

## Discussion

This study shows that women have a higher protein content of VLCAD, MCAD and TFPα than men, suggesting that women have a higher capacity for β-oxidation of LCFAs. Previous research has shown that there is a sex difference in FATm mRNA [17], FABPpm protein and mRNA, FAT/CD36 protein and mLPL [18] these results indicate that as compared with men, women have a higher capacity for fatty acid transport. Taken together, these results suggest women have a higher capacity than men for LCFA utilization; including skeletal muscle up-take and β-oxidation within mitochondria at rest and during exercise. These results are in agreement with our hypothesis and directly support previous observations that women oxidize more fat than men during endurance exercise [1]–[9], [31]–[33].

We conducted a comparative analysis by matching men and women based on their aerobic capacity expressed per unit fat-free mass (VO_2peak_·kg FFM^−1^·min^−1^). We [1], [6]–[8], [33]–[37] and others [2]–[3], [38] consistently match men and women using VO_2peak_ per kg FFM per min because endurance training is known to alter substrate utilization, increasing reliance on lipid sources and reducing the reliance on carbohydrates [5], [39]–[40]. Furthermore, we controlled for mitochondrial content by comparing CS protein content and observed no sex difference in total amount. Our data, taken together with previous findings that matched men and women have similar mitochondrial volume densities [34] and similar enzyme activities of CPT1 [24], 3-β-hydroxyacyl CoA dehydrogenase, citrate synthase, succinate-cytochrome c oxidoreductase, and cytochrome c oxidase [34], we believe our paired subjects to be well matched.

Women, having a higher abundance of VLCAD and MCAD than men, also showed a higher potential for utilization of straight-chain FAs. VLCAD and MCAD are two of the nine identified acyl-CoA dehydrogenases, and have broad chain-length enzyme activity raging from 4–16 carbon FA (MCAD) and 12–24 carbon (VLCAD) [41]. Trifunctional protein is a heterooctamer composed of 4α- and 4β-subunits. The alpha subunit, from the *HADHA* gene [42], has enzyme activity to hydrolyze long-chain enoyl-CoA and oxidize long-chain 3-hydroxyacyl-CoAs of 10 carbon chain-length and greater [43]. During exercise, FAs contribute anywhere from 30–70% of substrate utilized (depending on exercise intensity) [44], of which ∼90% are LCFAs [45]. The percentages of the predominant LCFA relative to the total plasma FFA are ∼43% oleate, ∼30% palmitate and ∼14% stearate [46]. This ratio is consistent at rest and does not change significantly during exercise [46] despite the large change in flux. However, LCFA kinetics in skeletal muscle do not reflect plasma concentrations, with women showing an increase in IMCL utilization during exercise compared with men [16]. Given the higher IMCL content, the close proximity of IMCL's to mitochondria [15] and the significantly greater protein abundance of VLCAD, MCAD and TFPα in women compared to men may help to explain the mechanism behind the observed sex difference in lipid oxidation during endurance exercise.

In order to elucidate the potential transcriptional mechanism regulating the increase protein content of VLCAD, MCAD and TFPα we looked at protein content of PPARα and PPARγ. Our data show no significant difference in the total amount of these transcription factors in whole muscle homogenate. Recent work from our lab showed that there is a greater constitutive expression of two of the genes encoding key modulators of fat oxidation (*PPARα* and *PPARδ*) in women compared with men [19]. PPARα is a transcriptional activator of *MCAD*, *CPTI*, *CPTII*, *FABP* and *FATP* [47]–[48], whereas PPARδ transcriptionally activates *FABPc*, *FAT/CD36*, *LPL*, *CPTI*, and genes involved in β-oxidation [49]–[51]. Less is known about the role of PPARγ in skeletal muscle. PPARγ has 2 splice varents; PPARγ1 and PPARγ2, and it is PPARγ1 that is expressed in skeletal muscle and has greater expression in oxidative fibers [52]. The most convincing evidence for PPARγ1's role in metabolism is that genetic skeletal muscle PPARγ1 knockout mice are less responsive to thiazolidinedione (a PPARγ inhibitor) treatment compared with wild-type controls, and were more susceptible to insulin resistance [53]. Furthermore, genetic activation of PPARγ1 in mouse skeletal muscle protected against high-fat diet-induced insulin resistance, reduced myocyte lipid content and improved muscle oxidative fibers, either directly or indirectly (through the release of adiponectin), increasing genes involved in lipid oxidation and mitochondria function [54]–[56]. Here we show that there is no significant difference in PPARα or PPARγ protein content between men and women. The lack of difference in protein content of PPARα despite differences in mRNA is consistent with similar findings with PPARδ [19]. Although changes in total protein content are not different between men and women, transcription factors rely on nuclear abundance and translocation into the nucleus to elicit changes in RNA expression. Future studies should include sex differences in nuclear abundance of these transcription factors. Also, we cannot negate that recent studies in skeletal muscle biology have also found discrepancies in the correlation between mRNA and protein content of a number of genes related to fatty acid oxidation [10], [18], [57]. Part of the discrepancy between mRNA abundance, protein content, and enzyme assays may be that multiple small changes in interacting and synergistic pathways/factors combine to influence flux through metabolic pathways at the protein level, and that each change is below the detectable threshold for statistical significance. It could also be the technical sensitivity of RT-PCR compared with the higher variance in Western blots and activity assay techniques. Regardless, in order to fully understand cellular differences between men and women it is important to understand pre-translational (mRNA abundance), translational (protein) and post-translational (phosphorylation, degradation) levels of control.

One of the possible contributing factors for the observed sex difference could be estrogen effects. Research using rodents [58]–[60] and humans [35]–[36], [61]–[62] has suggested that estrogen may play a role in regulating substrate utilization. The administration of 17β-estradiol to amenorrheic women [62] and to men [35]–[36], [61] lowers the respiratory exchange ratio during exercise [35]–[36], [61]–[62], reduces whole body carbohydrate and leucine oxidation. It also increases lipid oxidation in men [36], increases plasma FFA concentration [62] and reduces glucose rate of appearance [35], [61]–[62], rate of disappearance and metabolic clearance rate [35], [61]. In favor of a direct role for estrogen in sex differences in substrate utilization, database searches reveal that MCAD, SCHAD, PPARα, PPARδ, and PPARγ have up-stream estrogen response elements (ERE) [63] suggesting these genes may be regulated by estrogen.

In conclusion, our results show that women have higher VLCAD, MCAD, and TFPα protein than age and fitness matched men. Our findings offer an explanation for the observed sex differences in lipid oxidation. Taken together with the evidence that women have greater FA transport capabilities in skeletal muscle, we can conclude that women are more efficient at using lipid as a substrate during endurance exercise than men.
